# Platelet CLEC-2 activation leads to GPIb⍺ shedding: Implications for doxorubicin chemotherapy and thrombosis

**DOI:** 10.1016/j.jbc.2026.113074

**Published:** 2026-04-27

**Authors:** Zackary Rousseau, Wenjing Ma, Tianle Long, Sladjana Slavkovic, Xin Qiu, Xiaomei Lao, Xun Grace Wu, Kaishiv Joshi, Yunqing Amelia Zhu, Guangheng Zhu, Kelsie L. Thu, Heyu Ni

**Affiliations:** 1 Department of Laboratory Medicine and Pathobiology, University of Toronto, Toronto, Ontario, Canada; 2 Department of Laboratory Medicine, LKSKI-Keenan Research Centre for Biomedical Science, St Michael’s Hospital, Toronto, Ontario, Canada; 3 Toronto Platelet Immunobiology Group, Toronto, Ontario, Canada; 4 Canadian Blood Services Centre for Innovation, Toronto, Ontario, Canada; 5 Department of Physiology, University of Toronto, Toronto, Ontario, Canada; 6 Department of Medicine, University of Toronto, Toronto, Ontario, Canada

**Keywords:** ADAM17, CLEC-2, doxorubicin, GPIbα shedding, integrin αIIbβ3, platelets, thrombocytopenia, thrombosis

## Abstract

Doxorubicin (Dox) is a potent first-line chemotherapeutic and widely administered against different types of cancer, but is associated with a myriad of side effects, including cancer/chemotherapy-associated thrombosis and drug-induced thrombocytopenia. Although we and others have reported Dox-induced platelet activation, the binding partner of Dox on platelets has not been previously explored. Here, we found human and mouse platelet aggregation triggered *via* C-type lectin-like receptor-2 (CLEC-2) was obstructed by Dox, but aggregation induced by classical agonists like ADP, collagen/collagen-related peptide, or thrombin receptor-activating peptide 6, was unaffected. By isothermal titration calorimetry, we detected a high binding affinity between Dox and recombinant CLEC-2 at 4.2 ± 2.4 nM. Interestingly, we found significant GPIb⍺ shedding from human and mouse platelet surfaces following Dox treatment. Consistently, GPIb⍺ shedding was recapitulated following anti-CLEC-2 monoclonal antibody treatment. Using Piceatannol to selectively inhibit CLEC-2 intracellular signaling or the pan-matrix metalloproteinases inhibitor GM6001 rescued glycoprotein Ibα (GPIbα) from both Dox and CLEC-2 mAb-induced shedding. Using GI254023X or GW280264X to specifically inhibit ADAM10 or ADAMs10/17, respectively, revealed inhibition of ADAM10/17, but not ADAM10 exclusively, prohibited GPIbα shedding. Collectively, this implicates the classical sheddase of GPIbα, ADAM17. Thus, we pinpointed CLEC-2 as a binding partner for Dox on platelets and a novel pathway of ADAM17-mediated GPIb⍺ shedding *via* CLEC-2. These data not only provide insights into a mechanism of Dox-induced platelet activation, thrombosis, and drug-induced thrombocytopenia, but also reveal putative precision therapeutic approaches for Dox-treated patients and nominate CLEC-2 inhibition as a means to regulate thrombotic disease and/or bleeding disorders.

Platelets, anucleate blood cells abundant in circulation, have frequently been reported to contribute to the pathology of cancer development and progression, wherein tumor cells may recruit platelets to activate, degranulate, and aggregate around them (1, 2, 3). Doxorubicin (Dox) is a first-line anthracycline chemotherapy for multiple cancer types, including triple-negative breast cancer and some sarcomas. Despite being a powerful antineoplastic agent, it has considerable adverse effects, including several platelet-related side effects such as drug-induced thrombocytopenia (DIT) and venous thromboembolism (4, 5). We previously reported Dox-induced platelet activation, which potentiates platelet aggregation and risk of thrombosis (6). However, the mechanism has not been adequately explored.

Platelets play key roles in thrombosis and hemostasis (7, 8, 9, 10, 11). Prominent, abundant surface receptors glycoprotein (GP)Ibα (12, 13, 14) and integrin αIIbβ3 (15, 16, 17) are responsible for the initial tethering to exposed extracellular matrix proteins and mediating platelet aggregation, respectively. glycoprotein Ibα (GPIbα), of the GPIb-IX-V receptor complex, is recognized as the primary receptor for von Willebrand factor (VWF), and this receptor–ligand interaction is particularly important in thrombus generation under high shear conditions, such as in arterial thrombus formation. Notably, GPIbα is cleaved by the matrix metalloproteinase (MMP) A Disintegrin and Metalloproteinase (ADAM), ADAM17 (18). GPIbα shedding is postulated to serve as a modulator for prolonged activation and aggregation by reducing VWF-binding and regulating hemostasis, both of which rely in part upon GPIbα (19). Thus, careful regulation of receptor shedding may signify an intrinsic mechanism for balancing hemostasis and thrombosis.

C-type lectin-like receptor-2 (CLEC-2) is a transmembrane receptor containing a hemi-immunoreceptor tyrosine-based activation motif (hemITAM) on its intracellular tail that is expressed by a variety of cells, including platelets (20, 21). Spleen tyrosine kinase (SYK) is an effector protein that purportedly associates with ITAMs to induce platelet activation along an SYK–phospholipase C–PKC axis (20, 22, 23). We previously reported inhibition of SYK mitigated Dox-induced platelet activation, leading us to hypothesize Dox may activate platelets *via* CLEC-2 (6). Although CLEC-2 was initially recognized for its role in fetal development and thromboinflammation (24, 25), interestingly, recent discoveries have begun to shed light on a greater function of CLEC-2 in thrombosis (26, 27, 28, 29). This hints at a relationship between Dox, CLEC-2, and GPIbα in the broader scheme of thrombosis and hemostasis, yet this has never been previously explored.

Here, we demonstrate that Dox directly interacted with the platelet surface receptor CLEC-2, leading to MMP-mediated shedding of GPIbα. Intriguingly, CLEC-2 stimulation by anti-CLEC-2 mAb replicated this phenomenon, indicating a novel pathway of GPIbα shedding mediated by CLEC-2. These data describe a relationship between Dox, CLEC-2, and GPIbα, improving both our ability to guide Dox-treated cancer patient precision therapy to mitigate side effects and our understanding of CLEC-2 as a proposed antithrombotic target.

## Results

### Dox impeded CLEC-2-induced platelet aggregation *via* anti-CLEC-2 agonist binding

**Figure 1:**
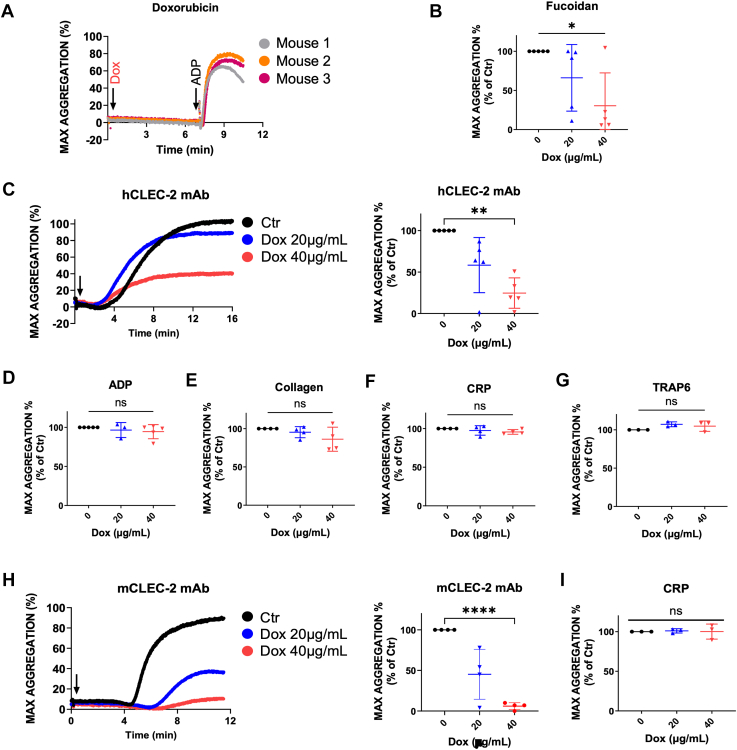
**Doxorubicin impeded CLEC-2-induced platelet aggregation *via* anti-CLEC-2 agonist binding.** Representative aggregation curve demonstrating no aggregation induced by Dox; ADP agonist control to stimulate normal platelet aggregation, assessed by light-transmission aggregometry (*A*). Human platelet-rich plasma (PRP) aggregation induced by the CLEC-2 agonist, Fucoidan, following ∼5 min Dox or vehicle control treatment (*B*). Representative aggregation curve and bar graph of human PRP aggregation induced by anti-CLEC-2 mAb agonist (*C*), or classical agonists ADP (*D*), Collagen/CRP (*E* and *F*), and TRAP6 (*G*) following Dox or vehicle control pretreatment. Representative aggregation curve and bar graph of mouse PRP aggregation induced by antimouse CLEC-2 mAb (*H*) or the traditional agonist, CRP (*I*). *n* = 3 to 5 for all assays. Values in bar graphs are displayed as mean ± SD. For comparison of multiple groups, a repeated measures one-way analysis of variance with multiple comparison tests was used on data normalized to respective controls within each experiment, where *p* < 0.05 (∗), *p* < 0.01 (∗∗), *p* < 0.0001 (∗∗∗∗), ns = not significant. CLEC-2, C-type lectin-like receptor 2; CRP, collagen-related peptide; Dox, doxorubicin; GPIbα, glycoprotein 1bα; IgG, immunoglobulin G; mAb, monoclonal antibody; PRP, platelet-rich plasma; TRAP6, thrombin receptor-activating peptide 6.

We previously reported Dox-induced platelet activation *via* the SYK–phospholipase C–PKC tyrosine kinase axis cascade (6). Due to SYK putatively being essential to CLEC-2-mediated activation (30, 31, 32, 33), yet the inability of Dox to directly induce aggregation (Fig. 1*A*), we assessed whether Dox instead blocked platelet aggregation mediated by CLEC-2. Human platelet aggregation induced by the CLEC-2 agonist, Fucoidan (34), was significantly reduced by 40 μg/ml Dox treatment (Fig. 1*B*). To look more specifically at the interaction of Dox and CLEC-2, we next observed anti-CLEC-2 mAb-induced aggregation, which similarly tended to be reduced at 20 μg/ml and was significantly impeded by ∼75% at 40 μg/ml Dox compared to vehicle control (Fig. 1*C*). We also tested other classic receptors that mediate platelet aggregation: P_2_Y_12_, GPVI, and PAR, using ADP, collagen and collagen-related peptide (CRP), or thrombin receptor-activating peptide 6, respectively. Platelet aggregation by these agonists was unimpeded by Dox (Fig. 1, *D*–*G*), indicating the specificity of Dox with CLEC-2.

Comparably, antimurine CLEC-2 mAb-induced mouse platelet aggregation tended to be reduced and was significantly impeded at 20 and 40 μg/ml Dox, respectively (Fig. 1*H*), whereas classical agonists such as CRP were unaffected (Fig. 1*I*). Collectively, these data suggest Dox bound to CLEC-2 and blocked aggregation-inducing ligand or antibody binding, but did not interact with other classical receptors on the platelet surface.

### Dox directly bound to recombinant CLEC-2

**Figure 2:**
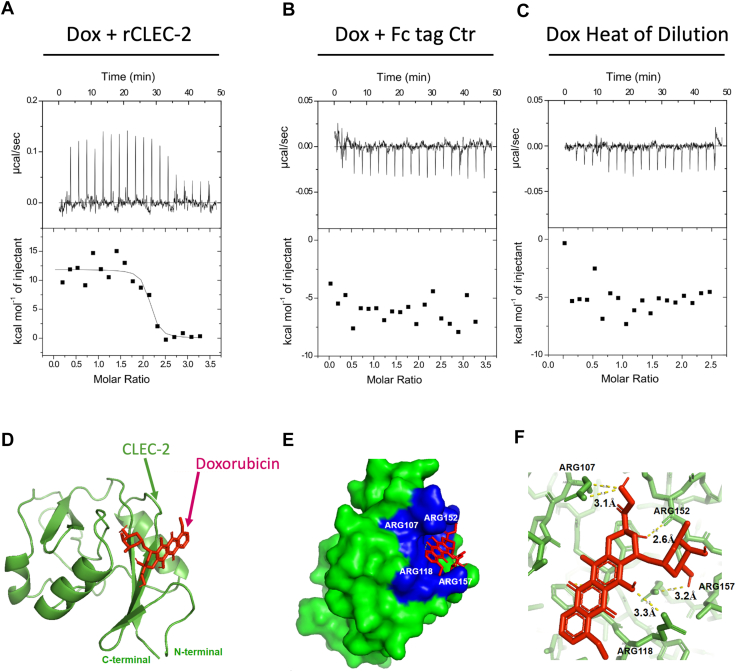
**Doxorubicin directly bound recombinant CLEC-2 protein.** ITC thermogram showing interaction between CLEC-2 and Dox (*A*); no interaction between Fc tag and Dox (*B*); heat of dilution of Dox (*C*). For each, on *top* is the raw titration data showing the heat resulting from each injection of ligand into buffer solution. The *bottom* shows the integrated heat plot after correcting for the heat of dilution. All experiments were performed at 25 °C in TB (pH 7.4). Binding affinity is reported as mean ± SD from two independent experiments. *In silico* docking prediction of the interaction between Dox and the extracellular region of CLEC-2 in cartoon structure (*D*). Surface view highlighting the docking of Dox and CLEC-2 with key interfacing Arg residues (*E*). Detailed representation of specific intermolecular bonds between Dox and surrounding CLEC-2 Arg residues, with hydrogen bonds depicted as *dashed lines* (*F*). Arg, arginine; CLEC-2, C-type lectin-like receptor 2; Dox, doxorubicin; H.o.D, heat of dilution; ITC, isothermal titration calorimetry; TB, Tyrode’s buffer.

To test whether direct interaction occurs between Dox and CLEC-2, we employed isothermal titration calorimetry (ITC) as we previously described (6, 35, 36, 37). The titration of Dox into recombinant CLEC-2 revealed a high-affinity interaction with an average binding affinity of 4.2 ± 2.4 nM quantified from independent titration replicates (Fig. 2*A*). To exclude Dox interaction with the Fc tag on the recombinant CLEC-2, an Fc tag-only control assay was performed, which displayed no interaction between Dox and Fc tag (Fig. 2*B*). In addition, no heat of dilution was detected for Dox alone (Fig. 2*C*), confirming that the observed change in thermodynamic signal was due to specific binding between CLEC-2 and Dox. Finally, under the conditions studied, these data revealed that Dox demonstrated a single binding site on CLEC-2, as the integrated ITC binding isotherm exhibited a single sigmoidal transition and was well described by a one-site binding model. The absence of additional inflection points further denotes that Dox interacts with CLEC-2 through a single binding site. In summary, characterized by an endothermic profile and single transition in the isotherm, the binding follows a one-site, entropy-driven mechanism.

Using *in silico* computational docking analyses to theoretically compute potential Dox-CLEC-2 binding sites, a predicted high-affinity interaction site was identified for Dox within the binding pocket of CLEC-2, coinciding with the interaction interface reported for its physiological ligands, podoplanin and the snake venom toxin, rhodocytin (Fig. 2*D*) (38). Notably, this pocket is comprised of key residues including Arg107, Arg118, Arg152, and Arg157, which form a positively charged cluster that facilitates strong electrostatic interactions with ligand molecules, while surrounding residues contribute through hydrogen bonding and hydrophobic contacts, facilitating the interaction between Dox and CLEC-2 (Fig. 2*E*). The predicted interaction distances between Dox and the Arg residues indicate strong to moderate binding affinity and stable, biologically relevant contact (Fig. 2*F*) (39). In summary, these data suggest Dox specifically binds to a single site on CLEC-2, likely in or around the ligand binding domain (40), which may be corroborated by the observed impediment of anti-CLEC-2 mAb-induced aggregation in Figure 1.

### Dox induced GPIbα shedding *in vitro*

**Figure 3:**
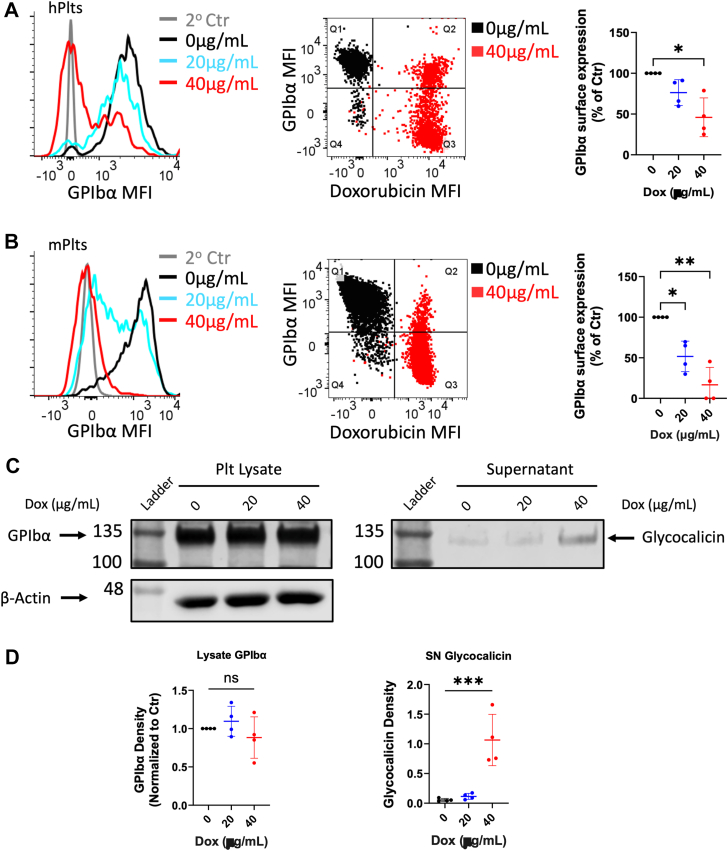
**Doxorubicin induced GPIbα shedding from platelets.** Surface GPIbα expression on platelets measured by flow cytometry following 4 h Dox treatment of human (*A*) and mouse (*B*) platelets. Data are represented by histogram (*left*), dot plot of GPIbα signal *versus* Dox autofluorescent signal depicting the correlation of Dox uptake and GPIbα decrease per platelet (*center*), and bar graph (*right*). *n* = 4. Representative blots (*C*) and quantification (*D*) of total supernatant and cell lysates from Dox-treated human platelets were assessed by Western blot using anti-glycocalicin antibody to detect the N terminus of membrane-bound or shed GPIbα in the lysate *versus* supernatant (*C*). *n* = 4. Positions of molecular mass markers indicated to the left of representative blots. Values in bar graphs are displayed as mean ± SD. For comparison of multiple groups, a repeated measures one-way analysis of variance with multiple comparison tests was used on data normalized to respective controls within each experiment, where *p* < 0.05 (∗), *p* < 0.01 (∗∗), *p* < 0.001 (∗∗∗), ns = not significant. 2^o^, secondary; Dox, doxorubicin; GPIbα, glycoprotein Ibα; hPlts, human platelets; MFI, median fluorescence intensity; mPlts, mouse platelets; SN, supernatant.

While attempting to characterize the effects of Dox on platelet activation, we observed GPIbα shedding, in line with previously described GPIbα shedding kinetics (41). Using flow cytometry, we detected a significant reduction in surface GPIbα expression of approximately 50% of vehicle control, on average, at 40 μg/ml Dox and a trending but insignificant reduction at 20 μg/ml Dox on human platelets after 4 h (Fig. 3*A*). Mouse platelets treated with Dox demonstrated more extensive surface GPIbα downregulation at both 20 μg/ml and 40 μg/ml Dox, with approximately 50% and 90% reduction in surface GPIbα expression, respectively (Fig. 3*B*).

To determine whether the downregulation of GPIbα is due to shedding or internalization, we probed Dox-treated platelet lysate and supernatant for glycocalicin (GC) *via* Western blot. GC is the ∼130 to 145 kDa N-terminal ectodomain portion released by cleavage at the juxtamembrane region of GPIbα (∼135–150 kDa) (42, 43, 44). We observed a significant increase of a ∼130 kDa fragment from the releasate of platelets treated with 40 μg/ml Dox, compared to the control and 20 μg/ml-treated groups (Fig. 3, *C* and *D*), indicating GC release from platelets. Thrombus formation under flow conditions of whole blood from mice injected with Dox was also tested in an *ex vivo* perfusion chamber model over immobilized collagen. Interestingly, compared to vehicle control mouse blood, platelet aggregation and thrombus growth from Dox-injected mouse blood trended to be reduced under high shear conditions of 1800 s^−1^ (Fig. S1*A*), but was indistinguishable under low shear conditions of 300 s^−1^ (Fig. S1*B*), intimating GPIbα shedding that mainly impaired arterial thrombosis at high shear (11, 13, 45, 46).

### Anti-CLEC-2 antibody-induced GPIbα shedding *in vitro*

**Figure 4:**
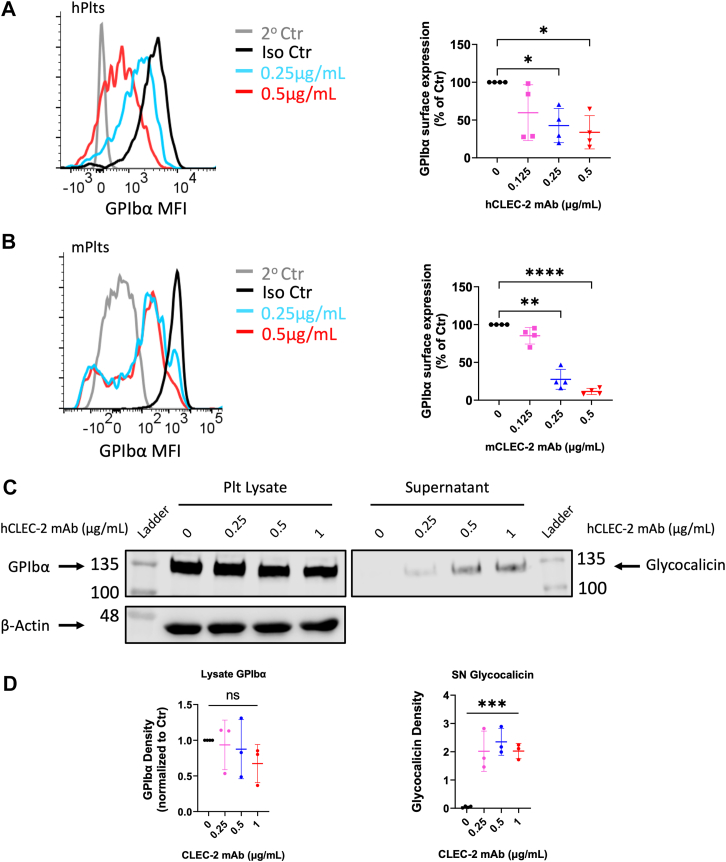
**Anti-CLEC-2 antibody-induced GPIbα shedding from platelets.** Surface GPIbα expression on platelets measured by flow cytometry following 4 h anti-CLEC-2 mAb treatment of human (*A*) and mouse (*B*) platelets. Data are represented by histogram (*left*) and bar graph (*right*). *n* = 4. Total supernatant and cell lysate fractions from human platelets treated with anti-CLEC-2 mAb were assessed by Western blot analysis using anti-glycocalicin antibody to probe the N terminus of GPIbα (*C*). Western blot densitometry quantification from lysates and supernatants (*D*). *n* = 3. Positions of molecular mass markers indicated on the *left* and *right* of the representative blots. Values in bar graphs are displayed as mean ± SD. For comparisons of multiple groups, a repeated measures one-way analysis of variance with multiple comparison tests was used on data normalized to respective controls within each experiment, where *p* < 0.05 (∗), *p* < 0.01 (∗∗), *p* < 0.0001 (∗∗∗∗), ns = not significant. 2^o^, secondary; CLEC-2, C-type lectin-like receptor 2; Ctr, control; Dox, doxorubicin; GPIbα, glycoprotein Ibα; hPlts, human platelets; IgG, immunoglobulin G; Iso, isotype; mAb, monoclonal antibody; mPlts, mouse platelets; SN, supernatant.

We next investigated whether the Dox-induced GPIbα shedding could be resultant from its interaction with CLEC-2. We observed a dose-dependent shedding of GPIbα by anti-CLEC-2 mAb treatment in human platelets, with significant decreases of GPIbα expression by ∼57% and 66% at 0.25 μg/ml and 0.5 μg/ml mAb concentration, respectively (Fig. 4*A*). Consistent with Dox-induced shedding, mouse platelets treated with anti-CLEC-2 mAb exhibited greater shedding response than human platelets: ∼75% at 0.25 μg/ml and 90% elimination of surface GPIbα expression at 0.5 μg/ml (Fig. 4*B*).

The supernatant and cell lysate of human platelets treated with anti-CLEC-2 mAb were collected and resolved by Western blot, then probed for GC. This revealed a trending decrease of GPIbα density in the lysate that was reflected in a significant corresponding increase of supernatant GC residue (Fig. 4, *C* and *D*).

### Anti-CLEC-2 antibody induced GPIbα shedding *in vivo*

**Figure 5:**
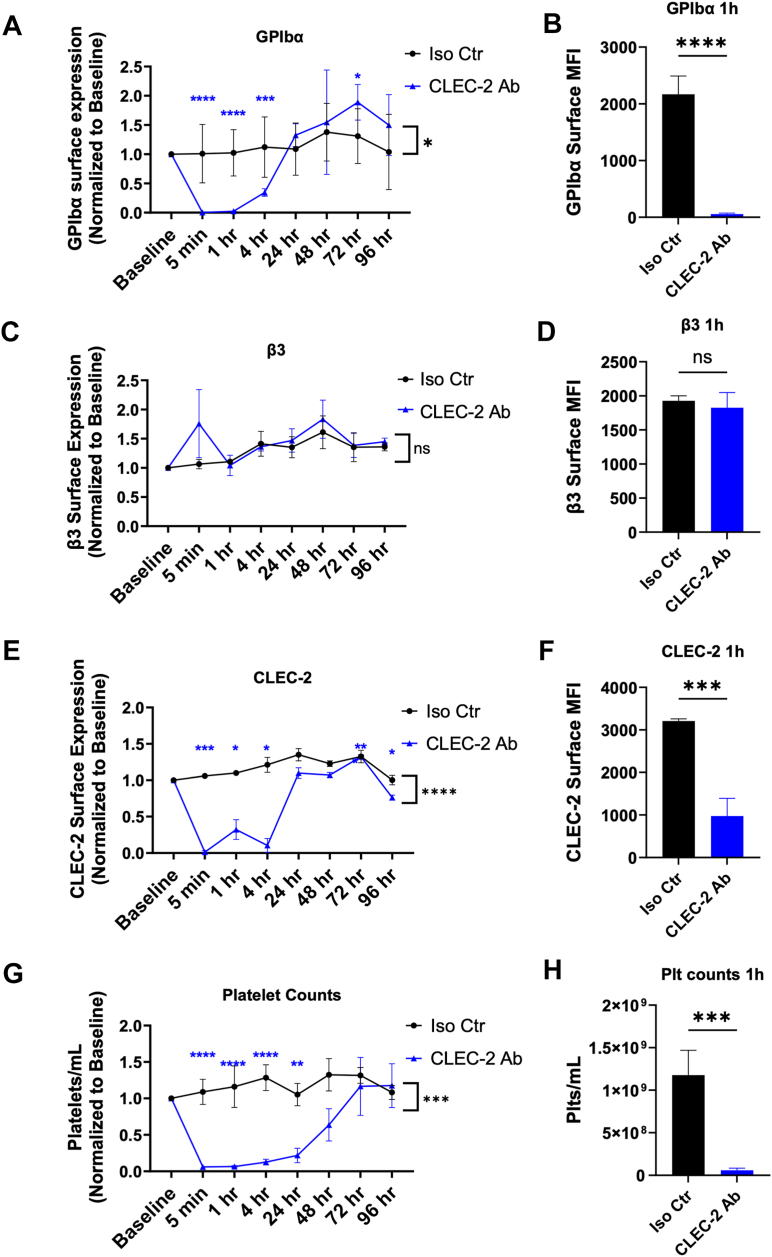
**Anti-CLEC-2 Antibody Induced GPIbα Shedding *in vivo*.** Time series analysis and representative direct comparison at 1 h for surface expression of GPIbα (*A* and *B*), integrin β3 (*C* and *D*), and CLEC-2 (*E* and *F*) from circulating platelets as well as platelet counts (*G* and *H*) in mice following a 0.4 μg/g IV dosage of anti-CLEC-2 mAb or isotype control IgG. Antibody or IgG control was injected and samples of circulating platelets were collected *via* saphenous bleeding at the respective time points then analyzed for the indicated receptor by flow cytometry. *n* = 4 to 6. Values are plotted as mean ± SD. A two-way ANOVA was performed to determine difference between treatment groups indicated by the *black bracket* and *asterisks*, while a repeated measures one-way ANOVA with multiple comparisons determined deviation from baseline and is indicated by the *blue asterisks* (*A*, *C*, *E*, and *G*), in both cases on data normalized to respective controls within each experiment. To compare difference in receptor expression at 1 h postinjection between isotype- and Ab-receiving groups, a two-tailed unpaired student’s *t* test was performed (*B*, *D*, *F*, and *H*). CLEC-2, C-type lectin-like receptor 2; GPIbα, glycoprotein Ibα; IgG, immunoglobulin G; Iso Ctr, isotype control; mAb, monoclonal antibody; Plt, platelet.

To determine whether CLEC-2-mediated GPIbα shedding occurred *in vivo*, mice were injected with a single 0.4 μg/g bolus of antimurine CLEC-2 mAb or 0.4 μg/g of rat IgG isotype control. As early as 5 min postinjection and through 1 h, GPIbα surface expression on circulating platelets was abolished in CLEC-2 mAb-injected mice with ∼100% reduction in GPIbα at the nadir (Fig. 5, *A* and *B*), while integrin β3 surface expression remained unchanged between the isotype control and Ab-injected cohorts (Fig. 5, *C* and *D*). Interestingly, GPIbα surface expression began to return to baseline within 24 h and exceeded basal GPIbα expression on day three before returning toward normal, which may have been a corrective response or variation from newly generated platelets (47, 48, 49).

In addition, immediately after CLEC-2 mAb injection, CLEC-2 expression on surviving platelets decreased significantly and began returning to baseline within 24 h (Fig. 5, *E* and *F*). Intriguingly, similar to GPIbα, CLEC-2 expression spiked briefly on day 3 before dropping off by day 4. As expected, platelet counts plummeted severely and remained thrombocytopenic until approximately day 3, where they returned to baseline levels (Fig. 5, *G* and *H*), consistent with previous reports of thrombocytopenia and CLEC-2 downregulation (50, 51). Examining platelets for residual CLEC-2 mAb postinjection revealed only a transient spike in surface-bound anti-CLEC-2 at 5 min, evidenced by the presence of rat IgG on the surface that quickly diminished to baseline (Fig. S2, *A* and *B*). This suggests that the observed decrease of CLEC-2 on circulating platelets was a result of downregulation rather than residual antibody blocking the epitope.

Anti-CLEC-2 mAb treatment induced GPIbα shedding from the surface of human and mouse platelets *in vitro* and *in vivo*, the latter leading to thrombocytopenia and reduced surface GPIbα and CLEC-2 for approximately 24 h. Collectively, these data indicate a novel mechanism of platelet GPIbα shedding mediated by CLEC-2 activation.

### Dox and anti-CLEC-2 antibody-induced GPIbα shedding attenuated by inhibiting CLEC-2 signal transduction and/or ADAM17 metalloproteinase activity, and is independent of αIIbβ3

**Figure 6:**
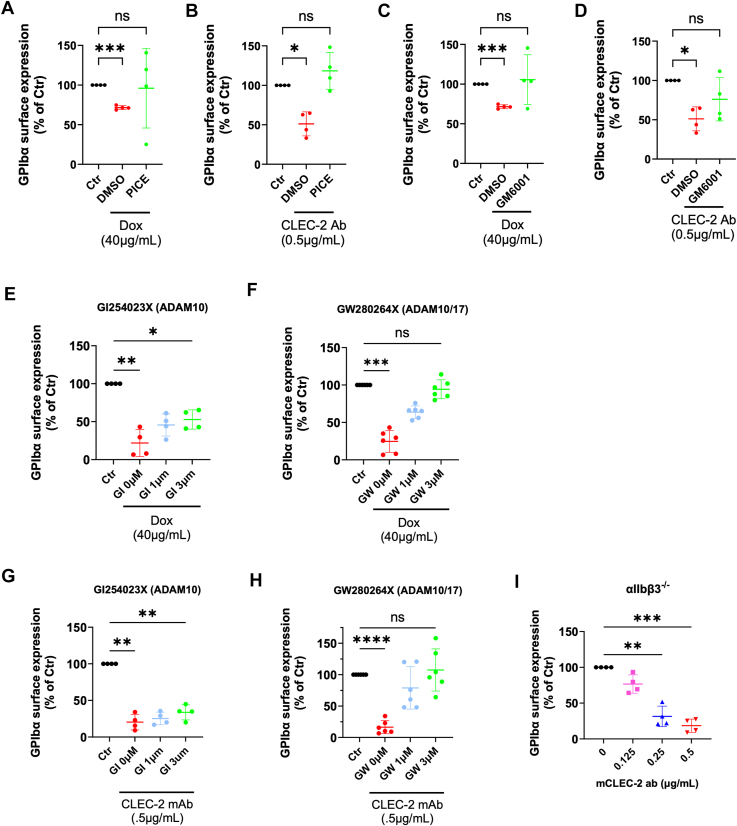
**Doxorubicin and CLEC-2 antibody-induced GPIbα shedding *via* SYK transduction was mediated by ADAM17 metalloproteinase, independent of integrin αIIbβ3.** Surface GPIbα expression on human platelets pre-treated with 300 μM Piceatannol measured by flow cytometry following 4 h Dox treatment (*A*) or anti-CLEC-2 mAb (*B*). Human platelets pre-treated with 100 μM GM6001 measured for GPIbα expression by flow cytometry following 4 h treatment with Dox (*C*) or anti-CLEC-2 mAb (*D*). Flow cytometric analysis for inhibition of Dox-induced GPIbα shedding on mouse platelets treated with ADAM10 specific inhibitor GI254023X (*E*) or ADAM10/17 selective inhibitor GW280264X (*F*). Analysis of inhibition for anti-CLEC-2 mAb-induced GPIbα shedding from mouse platelets by GI254023X (*G*) or GW280264X (*H*). Quantification of GPIbα surface expression following anti-CLEC-2 mAb-induced shedding on platelets from integrin αIIbβ3 genetic knockout mouse platelets (*I*). Data represented by bar graph as mean ± SD, *n* = 4 to 6 for all assays. For comparison of multiple groups, a repeated measures one-way analysis of variance with multiple comparison tests was used on data normalized to respective controls within each experiment, where *p* < 0.05 (∗), *p* < 0.01 (∗∗), *p* < 0.001 (∗∗∗), *p* < 0.0001 (∗∗∗∗), ns = not significant. CLEC-2 mAb, C-type lectin-like receptor 2 monoclonal antibody; Ctr, control; Dox, doxorubicin; GI, GI254023X; GPIbα, glycoprotein Ibα; GW, GW280264X; Pice, piceatannol.

Our results independently demonstrated that Dox directly interacted with CLEC-2 and Dox induced GPIbα shedding from platelets. To address whether Dox might induce GPIbα shedding through CLEC-2 activity, the selective small molecule inhibitor of SYK, Piceatannol, was used to blunt CLEC-2 signal transduction. Piceatannol treatment prevented GPIbα shedding on human platelets, as Dox treatment did not significantly reduce GPIbα expression compared to basal, vehicle control platelet GPIbα levels (Fig. 6*A*). Piceatannol similarly prevented GPIbα shedding induced by anti-CLEC-2 mAb (Fig. 6*B*); further evidencing a novel GPIbα shedding pathway mediated by CLEC-2 activation.

To determine whether the observed CLEC-2-mediated GPIbα shedding occurred by MMPs proteolytic cleavage, or some other mechanism unique to CLEC-2, human platelets were treated with the pan-MMP inhibitor GM6001. GM6001 rescued GPIbα expression in Dox-treated human platelets to levels comparable to untreated controls (Fig. 6*C*). GM6001 also abrogated GPIbα shedding by Dox in mouse platelets, seemingly more effectively than human (Fig. S3*A*). Human and mouse platelet GPIbα surface expression was similarly rescued by GM6001 against CLEC-2 mAb-induced shedding (Fig. 6*D*, Fig. S3*B*), pointing to the classical GPIbα sheddase ADAM17 (18) as the mechanism for CLEC-2-mediated GPIbα shedding.

To investigate ADAM17 specifically, mouse platelets were treated with either the specific ADAM10 inhibitor GI254023X (GI) or the ADAM10/17 inhibitor GW280264X (GW) prior to induction of GPIbα shedding by Dox or CLEC-2 mAb. Inhibition of ADAM10 by GI did not prevent GPIbα shedding induced by Dox (Fig. 6*E*), whereas inhibition of ADAM17 by GW rescued GPIbα expression (Fig. 6*F*). Consistently, GPIbα shedding induced by anti-CLEC-2 mAb was inhibited by GW, but not GI (Fig. 6, *G* and *H*). These data support a mechanism of ADAM17-mediated GPIbα shedding induced by Dox or CLEC-2.

In an attempt to further characterize the GPIbα shedding phenomenon induced by Dox and CLEC-2, we also observed dose-dependent CLEC-2 mAb-induced GPIbα shedding in αIIbβ3^−/−^ mice, indicating that the mechanism of CLEC-2-mediated GPIbα shedding by MMPs is independent of integrin β3 outside-in signaling on platelets (Fig. 4*I*) (52). Furthermore, we observed no change in platelet desialylation by Dox or CLEC-2 mAb (Fig. S4, *A* and *B*), which may be due to the GPIbα shedding as it contains ∼70% of sialylated residues on the platelet surface (53, 54, 55). In summary, these data suggest a novel and intriguing mechanism of GPIbα shedding by MMPs from the surface of platelets, likely mediated by CLEC-2 activation but independent of outside-in signaling through integrin αIIbβ3.

## Discussion

In this study, we identified platelet CLEC-2 as a binding partner for Dox. We further reveal a novel pathway of MMP-mediated surface GPIbα shedding following CLEC-2 signaling. These findings provide insight into Dox chemotherapy–associated thrombotic risk and deepen our understanding of the role of CLEC-2 in thrombosis and hemostasis.

We studied the possible mechanism and intracellular cascade following CLEC-2 stimulation leading to GPIbα downregulation on platelets. Initially, we hypothesized low-level/partial platelet activation prompted neuraminidase translocation to the platelet surface, resulting in desialylation of GPIbα leading to MMP-mediated GPIbα shedding and/or platelet clearance, as has been previously described (54, 56, 57, 58, 59). However, we observed no difference in platelet desialylation following Dox/anti-CLEC-2 mAb treatment. This would suggest CLEC-2-mediated GPIbα shedding occurs *via* an alternate mechanism. The rapid loss of GPIbα, CLEC-2 and circulating platelets following anti-CLEC-2 mAb injection (Fig. 5) is similar to kinetics described in a study reporting rapid CLEC-2 internalization induced by antibody or Fab fragments (50). If CLEC-2 associates with GPIbα, as has been previously postulated (26), it is possible that GPIbα may initially be internalized in conjunction with CLEC-2 by proximity, then recycled back to the platelet surface wherein shedding occurs along typical GPIbα shedding kinetics (41). An alternative explanation is that CLEC-2 activation modulates GPIbβ dissociation from the GPIb-IX-V complex, resulting in shedding of GPIbα by ADAM17, as previously reported (44). Nevertheless, although partial platelet activation *via* CLEC-2 by weak agonists like anti-CLEC-2 mAb or, even weaker, by Dox, may account for the prolonged time to GPIbα shedding observed *in vitro*, the disparity with rapid GPIbα shedding *in vivo* suggests a dynamic element of this mechanism that remains to be further investigated.

CLEC-2 activation *via* traditional ligands has been detailed intensively, yet nonclassical binding partners may reveal novel discoveries. It is largely agreed that, as CLEC-2 contains only a single YxxL hemITAM domain, dimerization and/or oligomerization is essential to localize the tandem hemITAM motifs for a sufficient magnitude of activation required to propagate CLEC-2-stimulated platelet aggregation (20, 32, 60). Our results demonstrate that Dox indeed binds strongly to CLEC-2, albeit with only a singular binding site. Together with the relatively miniscule size of Dox, this suggests Dox itself does not crosslink CLEC-2 monomers, which likely explains why we and others have reported that Dox does not directly induce aggregation (6, 61). These observations are consistent with a previous study reporting multimers that can crosslink CLEC-2 induced platelet aggregation, while in the absence of crosslinking, the nanobody served as an antagonist (62), further corroborating Dox impeded anti-CLEC-2 mAb-induced aggregation. Of note, this report also determined CLEC-2 expression levels directly correlated with the magnitude of activation response, as higher expression resulted in greater dimerization, which facilitated cross-linking (62). This may also explain the difference in magnitude between our human and mouse data, since mouse platelets with ∼20x greater CLEC-2 expression (40, 63) should experience a more potent effect by Dox. This is likewise consistent with our previous finding that Dox induced stronger activation in mouse platelets than human (6).

Although Dox cannot crosslink CLEC-2 to induce aggregation, Dox-induced platelet activation has been observed (61). The present ITC data and SYK-inhibition data (Figs. 2 and 6), as well as our previous observations of Dox-induced platelet activation, nonetheless suggest a mechanism of Dox-induced platelet activation occurring through CLEC-2. An early study described a mechanism of CLEC-2 translocation to lipid rafts following ligand binding in order to facilitate dimerization/oligomerization, and that this translocation is essential for hemITAM phosphorylation and signal initiation (64). Furthermore, previous findings report the presence of CLEC-2 dimers and oligomers basally present on resting platelets (65). Another study has previously described differential intracellular signaling and SYK engagement mechanisms depending on the magnitude of CLEC-2 receptor crosslinking (66). It is then altogether conceivable that Dox may “subliminally” or partially activate platelets *via* its interaction with CLEC-2, below the requisite threshold for aggregation.

It is not unprecedented that CLEC-2 may be “subliminally” or partially activated by a small molecule in the absence of crosslinking: a prior study reported anti-CLEC-2 Fab fragments impeded aggregation, yet still induced CLEC-2 downregulation and thrombocytopenia (67). In accordance with the findings of that study, we also observed thrombocytopenia and antibody-induced downregulation of CLEC-2, plus newly identified GPIbα shedding following CLEC-2 mAb injection *in vivo*, however, the duration of CLEC-2 downregulation was significantly shorter. This may be due to either or both our significantly lower dosage of antibody or an epitope-dependent disparity. It may be worthwhile to determine if CLEC-2-mediated GPIbα shedding is epitope dependent, which could influence targeting of CLEC-2 for antithrombotic development. The Fc-independent thrombocytopenia described in that study may be explained by CLEC-2-mediated GPIbα shedding, which is associated with platelet clearance (43, 68). The consequence of CLEC-2-mediated GPIb⍺ shedding inducing thrombocytopenia may be important to consider when pursuing CLEC-2 antithrombotic strategies.

The connection between CLEC-2 and GPIbα shedding has wider implications for thrombocytopenia commonly observed in patients receiving Dox. In our previous study, we suggested Dox potentiates platelet clearance dually by activation-induced senescence as well as enhancing clearance by phagocytosis, which could contribute to the observed risk of DIT associated with Dox (6). Interestingly, Dox-induced GPIbα shedding may have a compounded effect resulting in DIT, by directly causing platelet clearance as well as limiting platelet regeneration. Our prior work demonstrated GPIbα is an essential factor of platelet-mediated thrombopoietin synthesis, which is the hormone responsible for platelet production (69). Shedding of GPIbα may hinder physiological thrombopoietin regulation, contributing to the DIT sequelae in Dox-treated patients.

A recent study has distinguished two subpopulations of GPIbα, the surface-expressed protein as well as a greater subpopulation expressed intracellularly, the latter of which is postulated to be the substrate for the majority of released GC (41). Interestingly, our data indicate that CLEC-2 activation induces MMP-mediated GPIbα shedding from the surface population, though we cannot exclude contributions from intracellular granules or open canalicular system releasing GC as well. Although GC release and concentration in plasma has been postulated to serve a range of functions and as a marker for general platelet senescence or dysfunction (70, 71, 72), the endogenous physiological significance of GC is still undefined. Given that GC comprises nearly the entirety of extracellular GPIbα and therefore its binding site(s) (irrespective of other 60- and 45-kDa GC fragments that also contain the N-terminal VWF/thrombin binding head (73, 74)), GC release may impair platelet adhesion and aggregation. Furthermore, GPIbα is known to bind thrombin and facilitate hemostasis (75, 76, 77). Soluble GC has been demonstrated to control platelet aggregation induced by thrombin (78). Moreover, CLEC-2 is recognized for its role in thromboinflammation and, along with its ligand(s), serves to bridge the communication between platelets and leukocytes (79). It is conceivable that CLEC-2 induction of glycoprotein shedding, both GPVI as reported previously (40) and GPIbα as discussed in this report, by ADAMS10/17 serves to reduce the adhesion and activation of platelets *via* two of the prominent extracellular matrix-binding receptors to mitigate thrombus formation at sites of inflammation. Collectively, these patterns may reflect a physiological example of self-regulation to prevent pathological platelet aggregation and the formation of thrombi, as has previously been postulated, and may be an interesting topic for deeper exploration.

Previous studies have also reported impaired hemostasis and reduced thrombotic progression under arterial conditions in mice treated with CLEC-2-depleting antibodies at day 5 postdepletion (67, 80). These findings would seem to align with the proposed mechanism of CLEC-2-mediated GPIbα shedding affecting arterial thrombus growth and stability described herein; however consensus between our studies report GPIbα expression at or above basal levels by day 5. Although GPIbα is recognized as the classical receptor integral for thrombus growth in arterial shear conditions, it is evident that the interrelation between CLEC-2 and hemITAM signaling with GPIbα is far more complex and worthy of future deliberation. Nonetheless, CLEC-2 modulation of GPIbα shedding may be an underlying mechanism for both impaired arterial thrombosis and hemostasis and CLEC-2-induced thrombocytopenia.

To summarize, the present study elucidates a mechanism of Dox-induced platelet activation *via* its direct interaction with the platelet receptor CLEC-2. Furthermore, we report a novel mechanism of CLEC-2-mediated GPIb⍺ shedding by metalloproteinase(s). Taken together, our data have revealed exciting insights into fundamental platelet physiology and advances our understanding of CLEC-2 as a potential antithrombotic target through its regulation of GPIbα.

## Experimental procedures

### Reagents

Doxorubicin hydrochloride was from Thermo Fisher Scientific (Cat. No: J64000MA). PAR-1 Selective Agonist (thrombin receptor-activating peptide 6) (Cat. No. SCP0237-5MG), ADP (Cat. No. A2754–100MG), GM6001 (Cat. No. 364205-1MG), and Piceatannol (Cat. No. 527948-1MG) were purchased from Millipore Sigma. Purified antihuman CLEC-2 (Cat. No: 372002), Purified antimouse CLEC-2 (Cat. No: 146102) antibody, rat IgG2b Isotype control (Cat. No: 400602), and recombinant mouse CLEC-2 (Cat. No. 551806) from BioLegend. Collagen (Cat. No. 385) was obtained from Chrono-Log Corp. CRP-A was purchased from PPlusProducts. GI254023X (Cat. No: S8660) and GW280264X (Cat. No: E1012) were purchased from Selleckchem.

### Human and mouse blood samples

This study was approved by the Unity Health Toronto Research Ethics Board under approval number 22-004 on May 30, 2022. The research was conducted in accordance with the ethical principles outlined in the Declaration of Helsinki. All procedures involving human participants were carried out following a thorough risk-benefit analysis. Informed consent was obtained from all participants, and measures were implemented to ensure their privacy, confidentiality, and rights throughout the study.

The line of αIIb KO (αIIb^−/−^) mice was obtained from the laboratory of Dr Jon Frampton (Birmingham Medical School) (81), and was backcrossed onto C57BL/6 WT mice ten times. C57BL/6 WT mice, aged 6 to 8 weeks, were purchased from Charles River Laboratories. All mice were housed in the Research Vivarium of the Li Ka Shing Knowledge Institute (St Michael’s Hospital). All animal experiments were conducted in compliance with the Canadian Council on Animal Care and the St Michael’s Animal Care Committee guidelines, and were approved under protocol number ACC 359 on December 10, 2024 (previously ACC 166 approved November 16, 2021). Every effort was made to minimize animal suffering and to reduce the number of animals used.

### Preparation of human or mouse platelets

Human blood samples were collected into vacutainers containing 3.8% (*w/v*) sodium citrate from healthy volunteers. Platelet-rich plasma (PRP) was collected by centrifuging whole blood (300*g*, 7 min, ambient temperature, lowest brake). Washed platelets were prepared by diluting PRP with PBS-EDTA, then centrifuging (800*g*, 10 min, ambient temperature, no brake). The platelet pellet was resuspended in 1X Tyrode’s buffer (TB) [129 mM NaCl, 0.34 mM Na_2_HPO_4_, 2.9 mM KCl, 12 mM NaHCO_3_, 20 mM Hepes, 5 mM glucose, and 1 mM MgCl_2_ to a final pH of 7.4] to an approximate concentration of 3e8 platelets/ml. Murine blood was collected by retro-orbital bleeding into acid–citrate–dextrose solution A (Cat. No. 10357–1, Cepham Life Sciences, Inc,) at a ratio of 9:1. Mouse washed platelets were prepared in the same manner.

Human gel-filtered platelets was prepared as previously described (6, 82). Briefly, PRP was filtered through a Sepharose 2B (Sigma-Aldrich) column with TB and platelet fractions were collected.

### Platelet aggregation

Aggregation was performed as previously described (6, 46, 83, 84). Briefly, PRP was diluted in platelet-poor plasma or TB to a concentration of 2.5 × 10^8^ plts/ml and briefly incubated with Dox for ∼2 min pre-treatment. Aggregation was induced by respective agonists, and aggregation parameters measured in a light-transmission aggregometer (Model 700 Chrono-Log Corporation).

### ITC

ITC experiments were performed using a MicroCal ITC200 instrument (Malvern Panalytical) as we previously described (6, 35, 36, 37). Titrations were performed with mouse recombinant CLEC-2 (Fc-tagged) or Fc tag alone in the cell and Dox, as the titrant, in the syringe. All experiments were corrected for the heat of dilution of the titrant. Binding experiments consisted of an initial delay of 60 s, first injection of 0.2 μl and 150 s delay. Subsequent 19 injections were 2 μl, spaced every 150 s. The first point was removed from all data sets due to the different injection volume. The binding experiments were performed at 25 °C using CLEC-2 or Fc tag concentration of 2 μM and Dox concentration of 30 μM. ITC data were fit to a one-site binding model using the manufacturer-provided Origin 7 software.

### Computational docking

Molecular docking between CLEC2 protein and Dox was performed using CB-Dock2 online docking server, which automatically identifies putative binding cavities and performs blind docking within these regions. Experimentally determined three-dimensional structure of CLEC2 was retrieved from the Protein Data Bank (PDB ID: 2C6U; PDB, www.rcsb.org). The three-dimensional structure of the chemotherapeutic agent Dox (PubChem CID: 31703) was obtained from the PubChem database (www.pubchem.ncbi.nlm.nih.gov). The docking results were ranked based on Vina scores (kcal/mol), which represents docking score of the best interaction pose between CLEC-2 and Dox. The resulting docking complex of CLEC-2 and Dox was visualized and analyzed using PyMOL (Schrodinger, LLC) with structures rendered in cartoon and surface representation.

### Western blot of GPIbα

Human gel-filtered platelets were prepared, then treated with Dox or CLEC-2 mAb as indicated, or untreated as control, at 37 °C. Platelets were washed *via* centrifugation and the supernatant was collected. The platelet pellet was lysed using RIPA buffer (Cat. No. 89900, Thermo Fisher Scientific). Total protein from the lysate or supernatant were loaded and resolved by SDS-PAGE and Western Blot. The membrane was probed for GC (Cat. No. CAU25452, Biomatik Corp.) or Integrin β3 (in-house generated, mouse anti-integrin β3 mAb, PSI E1). Membranes were reprobed for β-actin-HRP (sc-47778 HRP, Santa Cruz Biotechnology) as the internal loading control. Imaging was performed by Li-Cor Odyssey Fc and densitometric analysis quantified using the Li-Cor Image Studio 6.1 software.

### Flow cytometry of GPIbα surface expression

Following treatment described in respective figures, platelets were then incubated with humanized anti-GPIbα fragment antigen-binding (Fab) antibody CA1001 (courtesy of CCOA Therapeutics Inc.), which was detected by antihuman kappa chain FITC-conjugated secondary antibody (Cat. No. 9230–02, Southern Biotechnology Associates). Platelet GPIbα expression was then measured by flow cytometry using a Sony SP6800 Spectral cytometer. Cytometry results were analyzed using FlowJo v10.8 software (BD Life Sciences).

### *In vivo* GPIbα shedding

WT c57BL/6 mice were intravenously injected with anti-CLEC-2 antibody. 10 μl whole blood samples were collected by pricking the saphenous vein into PBS-EDTA at the indicated time points. Platelets were isolated to measure GPIbα expression in the manner described above. Platelets were also stained with anti-β3 antibody conjugated with FITC (Cat. No: 553346, BD Pharminogen) and measured by flow cytometry. To detect CLEC-2 expression, platelets were incubated with antimouse CLEC-2 (BioLegend), and stained with antirat IgG conjugated with AlexaFluor-647 (Cat. No: 405416, Biolegend) for analysis by flow cytometry. Platelet counts were also performed using a Z2 series Coulter Counter (Beckman Coulter).

### *Ex vivo* perfusion chamber assays

*Ex vivo* perfusion assays were performed as previously described (46, 82, 85, 86). Briefly, rectangular microcapillary chambers (Ibidi GmbH) were coated with 100 μg/ml of collagen (Chrono-Log Corporation) overnight at 4 °C. Mice were injected with 10 μg/g of Dox, then murine whole blood was collected 24 h later from Dox-treated or control C57BL/6 mice *via* retro-orbital bleed with heparinized microcapillary tubes. The blood was fluorescently labeled using 1 μM DiOC6 for 5 mins on a rotator. Blood was perfused through the collagen-coated chamber using a syringe pump for ∼3 min at shear rates of 300 s^−1^ or 1800 s^−1^ for low and high shear, respectively. Images of platelet aggregation and thrombus growth were captured every 2 s *via* inverted fluorescence microscope (60x objective). Quantitative dynamics of median platelet fluorescence intensity were acquired by cellSens [vers 3.2] software (Olympus Corp.).

### Detection of platelet desialylation by flow cytometry

Following ∼30 min treatment with either Dox or anti-CLEC-2 mAb, human platelets were washed and incubated with Ricinus Communus Agglutinin −1 (RCA-1) conjugated with FITC (Cat. No: FL-1081–1, Vector Labs) to detect desialylated residues, as previously described (54, 87). Platelet desialylation was then measured by flow cytometry using a Sony SP6800 Spectral cytometer. Cytometry results were analyzed using FlowJo v10.8 software (BD Life Sciences).

### Statistical analysis

GraphPad Prism (version 10) was used for data visualization and analysis. Statistical analysis was performed using one-way ANOVA or Student’s *t* test accordingly. Results are presented as mean ± SD, with significance denoted as ∗ *p* < 0.05, ∗∗*p* < 0.01, ∗∗∗*p* < 0.001, and ∗∗∗∗*p* < 0.0001; ns = not significant.

## Data availability

All data described in this article are contained within.

## Supporting information

This article contains supporting information.

## Conflict of interest

The authors declare that they have no conflicts of interest with the contents of this article.

## References

[1] Xu X.R., Yousef G.M., Ni H. (2018). Cancer and platelet crosstalk: opportunities and challenges for aspirin and other antiplatelet agents. Blood.

[2] Xu X.R., Zhang D., Oswald B.E., Carrim N., Wang X., Hou Y. (2016). Platelets are versatile cells: new discoveries in hemostasis, thrombosis, immune responses, tumor metastasis and beyond. Crit. Rev. Clin. Lab. Sci..

[3] Wang S., Li Z., Xu R. (2018). Human cancer and platelet interaction, a potential therapeutic target. Int. J. Mol. Sci..

[4] Antoniak S., Phungphong S., Cheng Z., Jensen B.C. (2021). Novel mechanisms of anthracycline-induced cardiovascular toxicity: a focus on thrombosis, cardiac atrophy, and programmed cell death. Front. Cardiovasc. Med..

[5] Grover S.P., Hisada Y.M., Kasthuri R.S., Reeves B.N., Mackman N. (2021). Cancer therapy-associated thrombosis. Arterioscler Thromb. Vasc. Biol..

[6] Ma W., Rousseau Z., Slavkovic S., Shen C., Yousef G.M., Ni H. (2022). Doxorubicin-induced platelet activation and clearance relieved by salvianolic acid compound: novel mechanism and potential therapy for chemotherapy-associated thrombosis and thrombocytopenia. Pharmaceuticals (Basel).

[7] Ni H., Freedman J. (2003). Platelets in hemostasis and thrombosis: role of integrins and their ligands. Transfus. Apher. Sci..

[8] Roberts H.R., Hoffman M., Monroe D.M. (2006). A cell-based model of thrombin generation. Semin. Thromb. Hemost..

[9] Jackson S.P. (2011). Arterial thrombosis--insidious, unpredictable and deadly. Nat. Med..

[10] Wang Y., Ni H. (2016). Fibronectin maintains the balance between hemostasis and thrombosis. Cell Mol. Life Sci..

[11] Ruggeri Z.M. (2002). Platelets in atherothrombosis. Nat. Med..

[12] Savage B., Saldívar E., Ruggeri Z.M. (1996). Initiation of platelet adhesion by arrest onto fibrinogen or translocation on von Willebrand factor. Cell.

[13] Bergmeier W., Piffath C.L., Goerge T., Cifuni S.M., Ruggeri Z.M., Ware J., Wagner D.D. (2006). The role of platelet adhesion receptor GPIbalpha far exceeds that of its main ligand, von Willebrand factor, in arterial thrombosis. Proc. Natl. Acad. Sci. U. S. A..

[14] Ruggeri Z.M., Mendolicchio G.L. (2007). Adhesion mechanisms in platelet function. Circ. Res..

[15] Hodivala-Dilke K.M., McHugh K.P., Tsakiris D.A., Rayburn H., Crowley D., Ullman-Culleré M. (1999). Beta3-integrin-deficient mice are a model for Glanzmann thrombasthenia showing placental defects and reduced survival. J. Clin. Invest..

[16] Zou J., Swieringa F., de Laat B., de Groot P.G., Roest M., Heemskerk J.W.M. (2022). Reversible platelet integrin αIIbβ3 activation and thrombus instability. Int. J. Mol. Sci..

[17] Yang H., Reheman A., Chen P., Zhu G., Hynes R.O., Freedman J. (2006). Fibrinogen and von Willebrand factor-independent platelet aggregation *in vitro* and *in vivo*. J. Thromb. Haemost..

[18] Bergmeier W., Piffath C.L., Cheng G., Dole V.S., Zhang Y., von Andrian U.H., Wagner D.D. (2004). Tumor necrosis factor-alpha-converting enzyme (ADAM17) mediates GPIbalpha shedding from platelets *in vitro* and *in vivo*. Circ. Res..

[19] Baaten C., Swieringa F., Misztal T., Mastenbroek T.G., Feijge M.A.H., Bock P.E. (2018). Platelet heterogeneity in activation-induced glycoprotein shedding: functional effects. Blood Adv..

[20] Ozaki Y., Suzuki-Inoue K., Inoue O. (2013). Platelet receptors activated via mulitmerization: glycoprotein VI, GPIb-IX-V, and CLEC-2. J. Thromb. Haemost..

[21] Rayes J., Watson S.P., Nieswandt B. (2019). Functional significance of the platelet immune receptors GPVI and CLEC-2. J. Clin. Invest..

[22] Canobbio I., Bertoni A., Lova P., Paganini S., Hirsch E., Sinigaglia F. (2001). Platelet activation by von Willebrand factor requires coordinated signaling through thromboxane A2 and Fc gamma IIA receptor. J. Biol. Chem..

[23] Spalton J.C., Mori J., Pollitt A.Y., Hughes C.E., Eble J.A., Watson S.P. (2009). The novel Syk inhibitor R406 reveals mechanistic differences in the initiation of GPVI and CLEC-2 signaling in platelets. J. Thromb. Haemost..

[24] Lee R.H., Bergmeier W. (2016). Platelet immunoreceptor tyrosine-based activation motif (ITAM) and hemITAM signaling and vascular integrity in inflammation and development. J. Thromb. Haemost..

[25] Bergmeier W., Stefanini L. (2013). Platelet ITAM signaling. Curr. Opin. Hematol..

[26] Shao B., Hoover C., Shi H., Kondo Y., Lee R.H., Chen J. (2022). Deletion of platelet CLEC-2 decreases GPIbalpha-mediated integrin alphaIIbbeta3 activation and decreases thrombosis in TTP. Blood.

[27] Suzuki-Inoue K., Inoue O., Ding G., Nishimura S., Hokamura K., Eto K. (2010). Essential in vivo roles of the C-type lectin receptor CLEC-2: embryonic/neonatal lethality of CLEC-2-deficient mice by blood/lymphatic misconnections and impaired thrombus formation of CLEC-2-deficient platelets. J. Biol. Chem..

[28] Payne H., Ponomaryov T., Watson S.P., Brill A. (2017). Mice with a deficiency in CLEC-2 are protected against deep vein thrombosis. Blood.

[29] Sun L., Wang Z., Liu Z., Mu G., Cui Y., Xiang Q. (2024). C-type lectin-like receptor 2: roles and drug target. Thromb. J..

[30] Suzuki-Inoue K., Fuller G.L.J., García A., Eble J.A., Pöhlmann S., Inoue O. (2006). A novel syk-dependent mechanism of platelet activation by the C-type lectin receptor CLEC-2. Blood.

[31] Séverin S., Pollitt A.Y., Navarro-Nuñez L., Nash C.A., Mourão-Sá D., Eble J.A. (2011). Syk-dependent phosphorylation of CLEC-2: a novel mechanism of hem-immunoreceptor tyrosine-based activation motif signaling. J. Biol. Chem..

[32] Martyanov A.A., Balabin F.A., Dunster J.L., Panteleev M.A., Gibbins J.M., Sveshnikova A.N. (2020). Control of platelet CLEC-2-Mediated activation by receptor clustering and tyrosine kinase signaling. Biophys. J..

[33] Kostyak J.C., Mauri B., Dangelmaier C., Vari H.R., Patel A., Wright M. (2022). Phosphorylation on Syk Y342 is important for both ITAM and hemITAM signaling in platelets. J. Biol. Chem..

[34] Manne B.K., Getz T.M., Hughes C.E., Alshehri O., Dangelmaier C., Naik U.P. (2013). Fucoidan is a novel platelet agonist for the C-type lectin-like receptor 2 (CLEC-2). J. Biol. Chem..

[35] Ma X., Liang J., Zhu G., Bhoria P., Shoara A.A., MacKeigan D.T. (2023). SARS-CoV-2 RBD and its variants can induce platelet activation and clearance: implications for antibody therapy and vaccinations against COVID-19. Research (Wash D C).

[36] Neves M.A.D., Ni T.T., Mackeigan D.T., Shoara A.A., Lei X., Slavkovic S. (2024). Salvianolic acid B inhibits thrombosis and directly blocks the thrombin catalytic site. Res. Pract. Thromb. Haemost..

[37] Shoara A.A., Slavkovic S., Neves M.A.D., Bhoria P., Prifti V., Chen P. (2025). Structural analyses of apolipoprotein A-IV polymorphisms Q360H and T347S elucidate the inhibitory effect against thrombosis. J. Biol. Chem..

[38] Nagae M., Morita-Matsumoto K., Kato M., Kaneko M.K., Kato Y., Yamaguchi Y. (2014). A platform of C-type lectin-like receptor CLEC-2 for binding O-glycosylated podoplanin and nonglycosylated rhodocytin. Structure.

[39] McDonald I.K., Thornton J.M. (1994). Satisfying hydrogen bonding potential in proteins. J. Mol. Biol..

[40] Gitz E., Pollitt A.Y., Gitz-Francois J.J., Alshehri O., Mori J., Montague S. (2014). CLEC-2 expression is maintained on activated platelets and on platelet microparticles. Blood.

[41] Six K.R., Debaene C., Van den Hauwe M., De Rycke R., Gardiner E.E., Compernolle V., Feys H.B. (2023). GPIbalpha shedding in platelets is controlled by strict intracellular containment of both enzyme and substrate. J. Thromb. Haemost..

[42] Clemetson K.J., Naim H.Y., Lüscher E.F. (1981). Relationship between glycocalicin and glycoprotein Ib of human platelets. Proc. Natl. Acad. Sci. U. S. A..

[43] Bergmeier W., Burger P.C., Piffath C.L., Hoffmeister K.M., Hartwig J.H., Nieswandt B., Wagner D.D. (2003). Metalloproteinase inhibitors improve the recovery and hemostatic function of in vitro-aged or -injured mouse platelets. Blood.

[44] Mo X., Nguyen N.X., Mu F.T., Yang W., Luo S.Z., Fan H. (2010). Transmembrane and trans-subunit regulation of ectodomain shedding of platelet glycoprotein ibalpha. J. Biol. Chem..

[45] Ni H., Denis C.V., Subbarao S., Degen J.L., Sato T.N., Hynes R.O., Wagner D.D. (2000). Persistence of platelet thrombus formation in arterioles of mice lacking both von Willebrand factor and fibrinogen. J. Clin. Invest..

[46] Lei X., Reheman A., Hou Y., Zhou H., Wang Y., Marshall A.H. (2014). Anfibatide, a novel GPIb complex antagonist, inhibits platelet adhesion and thrombus formation in vitro and in vivo in murine models of thrombosis. Thromb. Haemost..

[47] Denis M.M., Tolley N.D., Bunting M., Schwertz H., Jiang H., Lindemann S. (2005). Escaping the nuclear confines: signal-dependent pre-mRNA splicing in anucleate platelets. Cell.

[48] Yang H., Lang S., Zhai Z., Li L., Kahr W.H.A., Chen P. (2009). Fibrinogen is required for maintenance of platelet intracellular and cell-surface P-selectin expression. Blood.

[49] Zimmerman G.A., Weyrich A.S. (2008). Signal-dependent protein synthesis by activated platelets: new pathways to altered phenotype and function. Arterioscler Thromb. Vasc. Biol..

[50] Lorenz V., Stegner D., Stritt S., Vögtle T., Kiefer F., Witke W. (2015). Targeted downregulation of platelet CLEC-2 occurs through syk-independent internalization. Blood.

[51] Brown H.C., Beck S., Navarro S., Di Y., Soriano Jerez E.M., Kaczmarzyk J. (2023). Antibody-mediated depletion of human CLEC-2 in a novel humanized mouse model. Blood Adv..

[52] Rabie T., Strehl A., Ludwig A., Nieswandt B. (2005). Evidence for a role of ADAM17 (TACE) in the regulation of platelet glycoprotein V. J. Biol. Chem..

[53] Gröttum K.A., Solum N.O. (1969). Congenital thrombocytopenia with giant platelets: a defect in the platelet membrane. Br. J. Haematol..

[54] Li J., van der Wal D.E., Zhu G., Xu M., Yougbare I., Ma L. (2015). Desialylation is a mechanism of Fc-independent platelet clearance and a therapeutic target in immune thrombocytopenia. Nat. Commun..

[55] Quach M.E., Li R. (2020). Structure-function of platelet glycoprotein Ib-IX. J. Thromb. Haemost..

[56] Jansen A.J., Josefsson E.C., Rumjantseva V., Liu Q.P., Falet H., Bergmeier W. (2012). Desialylation accelerates platelet clearance after refrigeration and initiates GPIbα metalloproteinase-mediated cleavage in mice. Blood.

[57] Butta N., van der Wal D.E. (2025). Desialylation by neuraminidases in platelets, kiss of death or bittersweet?. Curr. Opin. Hematol..

[58] Wang Y. (2021). Desialylation of. Haematologica.

[59] Tao L., Zeng Q., Li J., Xu M., Wang J., Pan Y. (2017). Platelet desialylation correlates with efficacy of first-line therapies for immune thrombocytopenia. J. Hematol. Oncol..

[60] Watson A.A., Christou C.M., James J.R., Fenton-May A.E., Moncayo G.E., Mistry A.R. (2009). The platelet receptor CLEC-2 is active as a dimer. Biochemistry.

[61] Kim S.H., Lim K.M., Noh J.Y., Kim K., Kang S., Chang Y.K. (2011). Doxorubicin-induced platelet procoagulant activities: an important clue for chemotherapy-associated thrombosis. Toxicol. Sci..

[62] Clark J.C., Martin E.M., Morán L.A., Di Y., Wang X., Zuidscherwoude M. (2023). Divalent nanobodies to platelet CLEC-2 can serve as agonists or antagonists. Commun. Biol..

[63] Dunster J.L., Unsworth A.J., Bye A.P., Haining E.J., Sowa M.A., Di Y. (2020). Interspecies differences in protein expression do not impact the spatiotemporal regulation of glycoprotein VI mediated activation. J. Thromb. Haemost..

[64] Pollitt A.Y., Grygielska B., Leblond B., Désiré L., Eble J.A., Watson S.P. (2010). Phosphorylation of CLEC-2 is dependent on lipid rafts, actin polymerization, secondary mediators, and rac. Blood.

[65] Hughes C.E., Pollitt A.Y., Mori J., Eble J.A., Tomlinson M.G., Hartwig J.H. (2010). CLEC-2 activates Syk through dimerization. Blood.

[66] Dangelmaier C., Vari H.R., Wright M., Kostyak J.C., Kunapuli S.P. (2022). Clustering extent-dependent differential signaling by CLEC-2 receptors in platelets. Res. Pract. Thromb. Haemost..

[67] May F., Hagedorn I., Pleines I., Bender M., Vögtle T., Eble J. (2009). CLEC-2 is an essential platelet-activating receptor in hemostasis and thrombosis. Blood.

[68] Chen W., Liang X., Syed A.K., Jessup P., Church W.R., Ware J. (2016). Inhibiting GPIbα shedding preserves post-transfusion recovery and hemostatic function of platelets after prolonged storage. Arterioscler Thromb. Vasc. Biol..

[69] Xu M., Li J., Neves M.A.D., Zhu G., Carrim N., Yu R. (2018). GPIbα is required for platelet-mediated hepatic thrombopoietin generation. Blood.

[70] Beer J.H., Büchi L., Steiner B. (1994). Glycocalicin: a new assay--the normal plasma levels and its potential usefulness in selected diseases. Blood.

[71] Coller B.S., Kalomiris E., Steinberg M., Scudder L.E. (1984). Evidence that glycocalicin circulates in normal plasma. J. Clin. Invest..

[72] Debaene C., Feys H.B., Six K.R. (2024). Shedding light on GPIbα shedding. Curr. Opin. Hematol..

[73] Bergmeier W., Rackebrandt K., Schröder W., Zirngibl H., Nieswandt B. (2000). Structural and functional characterization of the mouse von Willebrand factor receptor GPIb-IX with novel monoclonal antibodies. Blood.

[74] Jandrot-Perrus M., Clemetson K.J., Huisse M.G., Guillin M.C. (1992). Thrombin interaction with platelet glycoprotein Ib: effect of glycocalicin on thrombin specificity. Blood.

[75] Okumura T., Jamieson G.A. (1976). Platelet glycocalicin: a single receptor for platelet aggregation induced by thrombin or ristocetin. Thromb. Res..

[76] Okumura T., Hasitz M., Jamieson G.A. (1978). Platelet glycocalicin. Interaction with thrombin and role as thrombin receptor of the platelet surface. J. Biol. Chem..

[77] Bendas G., Schlesinger M. (2022). The GPIb-IX complex on platelets: insight into its novel physiological functions affecting immune surveillance, hepatic thrombopoietin generation, platelet clearance and its relevance for cancer development and metastasis. Exp. Hematol. Oncol..

[78] Jamieson G.A., Okumura T., Hasitz M. (1979). Structure and function of platelet glycocalicin. Thromb. Haemost..

[79] Meng D., Luo M., Liu B. (2021). The role of CLEC-2 and its ligands in thromboinflammation. Front. Immunol..

[80] Bender M., May F., Lorenz V., Thielmann I., Hagedorn I., Finney B.A. (2013). Combined *in vivo* depletion of glycoprotein VI and C-type lectin-like receptor 2 severely compromises hemostasis and abrogates arterial thrombosis in mice. Arterioscler Thromb. Vasc. Biol..

[81] Emambokus N.R., Frampton J. (2003). The glycoprotein IIb molecule is expressed on early murine hematopoietic progenitors and regulates their numbers in sites of hematopoiesis. Immunity.

[82] Zhu G., Zhang Q., Reddy E.C., Carrim N., Chen Y., Xu X.R. (2017). The integrin PSI domain has an endogenous thiol isomerase function and is a novel target for antiplatelet therapy. Blood.

[83] Reheman A., Yang H., Zhu G., Jin W., He F., Spring C.M. (2009). Plasma fibronectin depletion enhances platelet aggregation and thrombus formation in mice lacking fibrinogen and von Willebrand factor. Blood.

[84] Shen C., Mackeigan D.T., Shoara A.A., Bhoria P., Zhu G., Karakas D. (2024). Novel GPIb-independent platelet aggregation induced by botrocetin: implications for diagnosis and antithrombotic therapy. J. Thromb. Haemost..

[85] Xu X.R., Wang Y., Adili R., Ju L., Spring C.M., Jin J.W. (2018). Apolipoprotein A-IV binds αIIbβ3 integrin and inhibits thrombosis. Nat. Commun..

[86] MacKeigan D.T., Yu S.Y., Chazot N., Zhang D., Khoury C.J., Lei X. (2024). Apolipoprotein A-IV polymorphisms Q360H and T347S attenuate its endogenous inhibition of thrombosis. Biochem. Biophys. Res. Commun..

[87] Li J., Karakas D., Xue F., Chen Y., Zhu G., Yucel Y.H. (2023). Desialylated platelet clearance in the liver is a novel mechanism of systemic immunosuppression. Research (Wash D C).

