# Supplementary material for: Platelet CLEC-2 activation leads to GPIb⍺ shedding: Implications for doxorubicin chemotherapy and thrombosis
Source: J Biol Chem. 2026 Apr 27;302(6):113074. doi: 10.1016/j.jbc.2026.113074 (PMC13223932; doi:10.1016/j.jbc.2026.113074)
Supplement: Supporting Information [file mmc1.docx]

Supporting Information

**Platelet CLEC-2 activation leads to GPIb⍺ shedding: implications for doxorubicin chemotherapy and thrombosis**

*Running title: Dox-induced platelet activation & GPIbα Shedding via CLEC-2*

Zackary Rousseau^1,2,3⸸^, Wenjing Ma^1,2,3⸸*^, Tianle Long^2,3^, Sladjana Slavkovic^1,2, 3, 4^, Xin Qiu^2,3^, Xiaomei Lao^2,3^, Xun Grace Wu^2,3^, Kaishiv Joshi^2,3^, Yunqing Amelia Zhu^2,3^, Guengheng Zhu^2,3^, Kelsie L. Thu^1,2^, Heyu Ni^1-6*^

^1^Department of Laboratory Medicine and Pathobiology, University of Toronto, Toronto, M5S 1A1, ON, Canada;

^2^Department of Laboratory Medicine, LKSKI-Keenan Research Centre for Biomedical Science, St. Michael’s Hospital, Toronto, M5B 1W8, ON, Canada;

^3^Toronto Platelet Immunobiology Group, Toronto, M5B 1W8, ON, Canada;

^4^Canadian Blood Services Centre for Innovation, Toronto, ON, M5G 2M1, Canada;

^5^Department of Physiology, University of Toronto, Toronto, ON, M5S 1A1, Canada;

^6^Department of Medicine, University of Toronto, Toronto, ON, M5S 1A1, Canada;

^⸸^These authors contributed equally to this work

^*^Correspondence:

*Heyu Ni, MD; Ph. D, FCAHS, Professor
Department of Laboratory Medicine and Pathobiology
Department of Medicine, and Department of Physiology University of Toronto
Senior Scientist of Canadian Blood Services Centre for Innovation
Platform Director for Hematology, Cancer and Immunological Diseases
St. Michael's Hospital

209 Victoria Street, Toronto, Ontario

CANADA   M5B 1W8

Email: Heyu.Ni@unityhealth.to

Wenjing Ma, Ph.D

Department of Laboratory Medicine,

LKSKI-Keenan Research Centre for Biomedical Science,

St. Michael's Hospital

209 Victoria Street, Toronto, Ontario

CANADA   M5B 1W8

**Figure S1 Dox-treated mice exhibited reduced ex vivo thrombus growth under high shear.** Thrombus formation in blood from mice treated with 10µg/g Dox after 24 hours appears to be impaired under high shear conditions (A), but not at low shear rates (B), when perfused over collagen-coated microcapillary chambers. *n* = 3-4 (A), *n* = 2-3 (B). Dox, doxorubicin; MFI, median fluorescence intensity; Ctr, control.

**Figure S2 Transient surface-bound anti-CLEC-2 antibody spike receded within 1 hour.** Time series analysis (A) and direct comparison at 1 hour (B) of residual surface-bound rat IgG (anti-CLEC-2 mAb) from circulating platelets following IV infusion of anti-CLEC-2 mAb or isotype control IgG. *n* = 4-6. Values are plotted as mean ± SD. GPIbα, glycoprotein Ibα; CLEC-2, c-type lectin-like receptor 2; mAb, monoclonal antibody; IgG, immunoglobulin G.

**Figure S3 Dox and CLEC-2 induced mouse platelet GPIbα shedding by MMPs.** Surface GPIbα expression on WT mouse platelets pre-treated with 100 µM GM6001 or DMSO vehicle control determined by flow cytometry following 4h Dox treatment (A) or anti-CLEC-2 mAb treatment (B). Data represented in bar graph as mean ± SD, *n* = 4. For comparison of multiple groups, a repeated measures one-way analysis of variance with multiple comparison tests was used on data normalized to respective controls within each experiment where p < 0.05 (*), p < 0.01 (**), ns = not significant. Ctr, control; WT, wild-type; Dox, doxorubicin; mCLEC-2 mAb, mouse c-type lectin-like receptor 2 monoclonal antibody; GPIbα, glycoprotein Ibα.

**Figure S4 Dox and CLEC-2 mAb did not induce detectable desialylation on human platelets.** Human platelet desialylation induced by 30 minute Dox (A) or CLEC-2 mAb (B) treatment detected by fluorescent RCA-1 binding to desialylated residues and measured by flow cytometry. Data represented in bar graph as mean ± SD, *n* = 3-4. For comparison of multiple groups, a repeated measures one-way analysis of variance with multiple comparison tests was used on data normalized to respective controls within each experiment where p < 0.05 (*), p < 0.01 (**), ns = not significant. RCA-1, Ricinus communis agglutinin I; MFI, median fluorescent intensity; Ctr, control; Dox, doxorubicin; CLEC-2 mAb, c-type lectin-like receptor 2 monoclonal antibody.
